# Vascular phenotypes in primary non-small cell lung carcinomas and matched brain metastases

**DOI:** 10.1038/bjc.2011.147

**Published:** 2011-05-03

**Authors:** A M Jubb, A Cesario, M Ferguson, M T Congedo, K C Gatter, F Lococo, A Mulè, F Pezzella

**Affiliations:** 1 Nuffield Department of Clinical Laboratory Sciences, University of Oxford, Level 4 – Academic Block, John Radcliffe Hospital, Headley Way, Headington, Oxford, OX3 9DU, UK; 2 Division of Thoracic Surgery, Department of Surgical Sciences, Catholic University, Rome, Italy; 3 IRCCS San Raffaele Pisana, Rome, Italy; 4 Department of Pathology, Catholic University, Rome, Italy

**Keywords:** vascular, angiogenesis, non-small cell lung carcinoma, brain, metastases

## Abstract

**Background:**

Anti-angiogenic therapy with bevacizumab (an anti-vascular endothelial growth factor (VEGF) antibody) predominantly targets immature blood vessels. Bevacizumab has shown a survival benefit in non-small cell lung carcinoma (NSCLC) and has recently been demonstrated to be safe in patients with brain metastases. However, it is not known whether bevacizumab is effective against brain metastases or whether metastases are representative of their primary in terms of VEGF expression, hypoxia, proliferation and vascular phenotype. The aim of this study was to evaluate these factors in a series of matched primary NSCLCs and brain metastases.

**Methods and Results:**

Immunohistochemistry showed strong correlation of carbonic anhydrase 9 expression (a marker of hypoxia) in primary and secondary cancers (*P*=0.0002). However, the proliferation index, VEGF expression, microvessel density and the proportion of mature vessels were discordant between primary and secondary cancers. The mean proportion of mature vessels was 63.2% higher in the brain metastases than the primary tumours (*P*=0.004). Moreover, the vascular pattern of the primary tumour was not representative of the metastasis.

**Conclusions:**

Brain metastases have a significantly higher proportion of mature vasculature, suggesting that they may be refractory to anti-VEGF therapy. These findings may have implications for clinical trials and biomarker studies evaluating anti-angiogenic agents in brain metastases.

Angiogenesis, the development of new blood vessels, is a feature common to many carcinomas and has been proposed as a hallmark of cancer (Hanahan and Weinberg, 2000). However, morphological assessment of lung carcinomas has revealed that they may be subdivided into angiogenic and relatively non-angiogenic tumours (Pezzella *et al*, 1997); the latter co-opt pre-existing vasculature to support an alveolar pattern of growth. Nevertheless, the majority of non-small cell lung carcinomas (NSCLCs) are angiogenic and express vascular endothelial growth factor-A (VEGF) (Jubb *et al*, 2004), the predominant pro-angiogenic ligand that is exploited by tumours (Kerbel, 2008). Animal models (Kim *et al*, 1993; Huang *et al*, 2004; Mancuso *et al*, 2006) and human studies (Willett *et al*, 2004) have shown that blocking VEGF signalling results in tumour shrinkage, associated with the pruning of angiogenic (proliferative, leaky and immature (i.e., without associated pericytes)) blood vessels. This observation led to the development of a humanised anti-VEGF monoclonal antibody, bevacizumab, which has shown an improved progression-free survival benefit in numerous tumour types (Hurwitz *et al*, 2004; Miller *et al*, 2007; Rini *et al*, 2010).

In recurrent or advanced NSCLC, the addition of bevacizumab to carboplatin and paclitaxel chemotherapy (E4599 study) resulted in a 2-month improvement in median overall survival (Sandler *et al*, 2006). When combined with cisplatin and gemcitabine (AVAiL study) in NSCLC, bevacizumab showed no improvement in overall survival, but demonstrated improved progression-free survival (Reck *et al*, 2010). The E4599 study excluded patients with brain metastases due to concerns that bevacizumab might exacerbate the incidence of tumour-associated brain haemorrhage (Sandler *et al*, 2006). Several analyses now suggest that bevacizumab is safe in patients with brain metastases (Socinski *et al*, 2009; Besse *et al*, 2010), but there is no evidence regarding the efficacy of bevacizumab in this indication. Indeed, there is little published on the vascular phenotype, VEGF expression, hypoxia and proliferation of NSCLC brain metastases, which may inform efficacy.

To date, no valid biomarkers of the survival benefit of bevacizumab have been identified. Objective response rates have been shown not to predict benefit from bevacizumab in colorectal cancer (Grothey *et al*, 2008), and there are concerns that changes in the MRI appearance of glioblastoma following bevacizumab treatment do not predict clinical benefit (Gerstner *et al*, 2010). Efforts to identify novel *in situ* biomarkers of efficacy have focused on available tissue samples from primary tumour resections (Ince *et al*, 2005; Jubb *et al*, 2006), to make inferences concerning the treatment of metastatic disease. However, while there is evidence to support this for metastatic colorectal cancer (Kuramochi *et al*, 2006), there is little evidence to suppose that this is a valid approach to inform the biology of NSCLC brain metastases.

In summary, a greater understanding of the vascular phenotype of NSCLC brain metastases may better inform the use of anti-angiogenic agents in this indication. The aim of this study was to assess the relative expression of VEGF, hypoxia, proliferation, microvessel density, vascular pattern and vascular maturity in a series of matched primary NSCLCs and brain metastases.

## MATERIALS AND METHODS

### Tissue

Formalin-fixed paraffin-embedded blocks for 15 cases of primary NSCLC with matched resected brain metastases were retrieved from the pathology archives at the Catholic University Hospital, Rome. Ethical approval for this research was granted by the local research ethics committees.

### Immunohistochemistry

Characterisation of the antibodies and immunohistochemical methods for VEGF (Turley *et al*, 1998), CA9 (Jubb *et al*, 2009), Ki67 (Sington *et al*, 2007), CD34 (Kuzu *et al*, 1992) and CD34 and smooth muscle actin (SMA) (double stain; QBEnd/10 and 1A4; Dako, Ely, UK) (Patel *et al*, 2006) have been described in detail elsewhere.

### Scoring

The maximum intensity of VEGF expression in >10% of tumour cells was recorded on a semiquantitative scale from 0 (no expression) to 3 (very strong expression). The percentage of tumour cells with membranous expression of CA9 was estimated by a pathologist. The percentage of tumour cells with nuclear positivity for Ki67 was counted by a pathologist. Microvessel density analysis was performed using the Chalkley method; at low power ( × 40), five vascular hotspots in CD34-labelled sections were selected. Microvessel density was assessed by counting the number of CD34-labelled vessels that overlapped with dots on a 25-point Chalkley eyepiece graticule in each high-power field ( × 200) (Fox *et al*, 1995). The five counts were then added together to provide the density score. Vascular maturity is defined by the coverage of endothelial cells (CD34 positive) by pericytes (SMA positive) and was calculated by counting the proportion of CD34-labelled vessels that were surrounded, at least in part, by SMA-labelled cells from 10 high-power fields ( × 400). Each case was classified according to the predominant vascular pattern (alveolar, basal, diffuse or papillary) described elsewhere (Pezzella *et al*, 1997). All scoring was performed blind to the patients’ identities.

### Statistical analyses

Student's *t*-test was used to assess the difference between subsets of continuous data and Spearman's correlation coefficient was used to assess covariance. The *χ*^2^ test was used to evaluate associations between categorical data and Cohen's *κ* statistic was used to determine agreement.

## RESULTS

**Figure 1:**
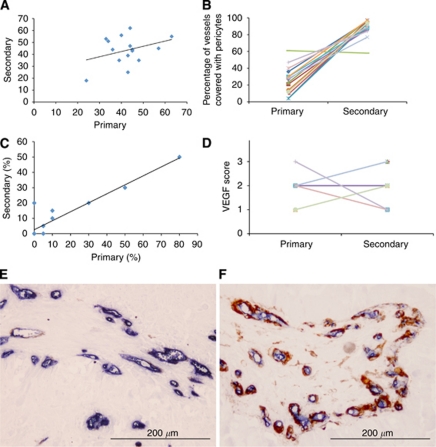
(**A**) A scatter plot of CD34 Chalkley counts in matched primary (lung) and secondary (brain) cancers. (**B**) A line plot showing differences in the percentage of blood vessels covered by pericytes in matched primary and secondary cancers. (**C**) A scatter plot of the percentage of matched primary and secondary cancers positive for carbonic anhydrase 9. (**D**) A line plot showing differences in the VEGF score in matched primary and secondary cancers. (**E**, **F**) Double-labelled immunohistochemistry for CD34 (blue) and smooth muscle actin (brown) in a matched primary (**E**) and secondary (**F**) cancer.

Chalkley counts in primary NSCLCs and brain metastases were not significantly correlated (*r*=0.148, *P*=0.60) (Table 1; Figure 1). Although, mean Chalkley counts were similar in primary and secondary cancers (42.5 *vs* 43.5, respectively, *P*=0.77). The proportion of mature vessels was on average 63.2% greater in brain metastases than their matched primary NSCLCs (mean 25.7% *vs* 88.9%, respectively, *P*=0.004) (Table 1; Figure 1). The proportions of mature vessels in primary and secondary cancers were not significantly correlated (*r*=−0.46, *P*=0.09). Differences were also observed in vascular patterns, with a predominance of alveolar, basal and diffuse patterns in primary tumours and a predominance of diffuse and papillary patterns in secondary tumours (Table 1).

The percentage of CA9-positive tumour cells was similar to that previously reported (Giatromanolaki *et al*, 2001) and was closely correlated between primary NSCLCs and brain metastases (*r*=0.825, *P*=0.0002), suggesting that there were similar levels of hypoxia in matched cases from this series (Table 1; Figure 1). The mean percentage of cells positive for CA9 was not statistically significantly different in primary and secondary cancers (14.0 *vs* 10.7, respectively, *P*=0.27). Vascular endothelial growth factor expression was seen in all primary and secondary NSCLCs; however, there was no agreement between the scores in matched pairs (*κ*=−0.25) and the association was not statistically significant (*P*=0.15) (Table 1; Figure 1). The proliferative fraction (percentage of Ki67-labelled cells) was also not significantly correlated between primary and secondary cancers (*r*=0.179, *P*=0.524), though the mean Ki67 was significantly higher in the brain metastases than the primary lung cancers (35.7% *vs* 19.3%, respectively, *P*=0.018) (Table 1).

## DISCUSSION

This is the first report comparing VEGF expression, CA9 expression, proliferation, microvessel density, vascular pattern and vascular maturity in matched primary NSCLCs and brain metastases. The data show that brain metastases have a significantly greater proliferation rate and vascular maturity than their matched primaries. Neither the proliferation rate, vascular maturity, expression of VEGF, vascular pattern nor microvessel density could be predicted in the brain metastasis from examination of the primary NSCLC. Only CA9 expression showed a strong correlation between primary and secondary NSCLCs. These observations are important for the following reasons:

First, anti-VEGF therapies were thought to increase the risk of cerebral haemorrhage in patients with brain metastases, but recent data suggest that bevacizumab is safe in this indication. Our data provide a biological explanation for this observation, showing that the vasculature of brain metastases is stable, and (extrapolating from observations in rectal cancer) (Willett *et al*, 2004) is unlikely to regress following anti-VEGF therapy, minimising the risk of associated haemorrhage.

Second, mature vasculature is less sensitive to anti-VEGF therapy, suggesting that while patients with primary NSCLCs may benefit from anti-VEGF therapy, those with brain metastases may not. Moreover, preclinical data suggest that when endothelial regression is seen in mature vessels targeted by anti-VEGF therapy, a pericyte scaffold remains and permits rapid re-angiogenesis following cessation of therapy (Mancuso *et al*, 2006). Thus, a rebound effect may be expected if anti-VEGF therapy is used in brain metastases and continuous, rather than intermittent, therapy might show greater efficacy. No efficacy data exist for bevacizumab in brain metastases, but Socinski *et al* (2009) have stated that ‘patients with treated brain metastases will likely derive similar benefit from bevacizumab as patients without brain metastases’ without any biological, pathological or clinical evidence. The data herein are an important counterbalance to this claim.

Third, non-angiogenic NSCLCs (Pezzella *et al*, 1997) that show an alveolar pattern of growth may metastasize, and do not merely co-opt existing vasculature in the brain, but instead develop a more angiogenic phenotype with haphazardly arranged vessels. Moreover, even non-angiogenic, alveolar tumours express VEGF, are under the influence of hypoxia and are supported by an often immature, dense vasculature. Therefore, one may expect non-angiogenic tumours to potentially respond to anti-VEGF therapy at their site of origin.

Finally, retrospective analyses of archived primary tumours from trials of anti-VEGF therapy have thus far failed to identify a biomarker of efficacy (Jubb and Harris, 2010). The data presented herein suggest that the vascular phenotypes of primary and secondary cancers are very different. Therefore, when evaluating anti-angiogenic treatments in the metastatic setting, biomarker studies should also be conducted on metastases and not the primary tumours.

The limitations of this study include the sample size, which is small in statistical terms, but it is large for a series of matched brain metastases, provides informative data and reports novel findings. Furthermore, while one may make inferences regarding the applicability of these observations to anti-VEGF therapy using preclinical data (Huang *et al*, 2004; Mancuso *et al*, 2006) and clinical data from other tumours (Willett *et al*, 2004), only serial biopsies of brain metastases from patients receiving bevacizumab will provide a definitive answer. To date, the two reported series of NSCLC brain metastases treated with bevacizumab in the literature are small, *n*=36 (Besse *et al*, 2010) and *n*=115 (Socinski *et al*, 2009) and provide no histopathological data. Moreover, it is unlikely that clinicians would want to subject terminally ill patients to repeated invasive procedures to provide such material.

In conclusion, brain metastases of NSCLCs have a vascular phenotype that is distinct from the primary tumours. This has implications for the use of anti-VEGF therapy in this setting and should be taken into consideration when designing clinical trials to assess the efficacy of such drugs and biomarkers that may predict benefit.

## Figures and Tables

**Table 1:** Scoring frequencies

**Case**	**VEGF intensity score (0 to 3)**		**CA9 (% positive)**		**Ki67 (% positive)**		**Chalkley count (*n*)**		**Vascular maturity (% mature)**		**Vascular pattern**	
**Site**	**Lung**	**Brain**	**Lung**	**Brain**	**Lung**	**Brain**	**Lung**	**Brain**	**Lung**	**Brain**	**Lung**	**Brain**
1	2	2	0	20	10	50	34	51	36	96	Basal	Papillary
2	2	1	0	0	50	50	45	43	21	88	Basal	Basal
3	2	3	0	0	10	30	39	35	23	97	Basal	Papillary
4	2	3	0	0	0	15	44	62	4	97	Alveolar	Papillary
5	2	2	30	20	60	80	44	47	4	93	Diffuse	Basal
6	1	2	5	0	20	15	45	44	14	95	Alveolar	Diffuse
7	2	3	0	0	40	30	33	53	27	94	Basal	Basal
8	2	2	10	15	0	70	24	18	9	58	Diffuse	Diffuse
9	2	2	0	0	40	40	57	45	61	86	Papillary	Papillary
10	2	1	0	0	0	20	40	56	30	88	Alveolar	Diffuse
11	1	2	30	20	10	5	43	25	30	97	Diffuse	Diffuse
12	2	3	80	50	30	10	63	55	28	77	Alveolar	Papillary
13	2	1	50	30	5	40	43	39	40	89	Basal	Diffuse
14	1	2	5	5	5	40	48	35	12	94	Diffuse	Papillary
15	3	1	0	0	10	40	36	44	47	84	Basal	Diffuse
Median	2	2	0	0	10	40	43	44	27	93	NA	NA
IQR	2–2	1.5–2.5	0–20	0–20	5–35	17.5–45	37.5–45	37–52	13–33	87–95.5	NA	NA

Abbreviations: CA9=carbonic anhydrase 9; IQR=interquartile range; NA=not applicable; VEGF=vascular endothelial growth factor.
